# Delivering Behaviour Change Interventions: Development of a Mode of Delivery Ontology

**DOI:** 10.12688/wellcomeopenres.15906.2

**Published:** 2021-02-26

**Authors:** Marta M. Marques, Rachel N. Carey, Emma Norris, Fiona Evans, Ailbhe N. Finnerty, Janna Hastings, Ella Jenkins, Marie Johnston, Robert West, Susan Michie

**Affiliations:** 1 Centre for Behaviour Change, University College London, London, UK; 2 ADAPT SFI Research Centre, Trinity College Dublin, Dublin, Ireland; 3 Trinity Centre for Healthcare and Practice Innovation, Trinity College Dublin, Dublin, Ireland; 4 Aberdeen Health Psychology Group, University of Aberdeen, Aberdeen, Scotland, UK; 5 Research Department of Epidemiology & Public Health, University College London, London, UK

**Keywords:** ontology, intervention, behaviour, reporting, expert feedback, evidence synthesis, delivery

## Abstract

**Background:** Investigating and improving the effects of behaviour change interventions requires detailed and consistent specification of all aspects of interventions. An important feature of interventions is the way in which these are delivered, i.e. their mode of delivery. This paper describes an ontology for specifying the mode of delivery of interventions, which forms part of the Behaviour Change Intervention Ontology, currently being developed in the Wellcome Trust funded Human Behaviour-Change Project.

**Methods:** The Mode of Delivery Ontology was developed in an iterative process of annotating behaviour change interventions evaluation reports, and consulting with expert stakeholders. It consisted of seven steps: 1) annotation of 110 intervention reports to develop a preliminary classification of modes of delivery; 2) open review from international experts (n=25); 3) second round of annotations with 55 reports to test inter-rater reliability and identify limitations; 4) second round of expert review feedback (n=16); 5) final round of testing of the refined ontology by two annotators familiar and two annotators unfamiliar with the ontology; 6) specification of ontological relationships between entities; and 7) transformation into a machine-readable format using the Web Ontology Language (OWL) and publishing online.

**Results:** The resulting ontology is a four-level hierarchical structure comprising 65 unique modes of delivery, organised by 15 upper-level classes: Informational *,* Environmental change, Somatic, Somatic alteration, Individual-based/ Pair-based /Group-based, Uni-directional/Interactional, Synchronous/ Asynchronous, Push/ Pull, Gamification, Arts feature. Relationships between entities consist of *is_a*. Inter-rater reliability of the Mode of Delivery Ontology for annotating intervention evaluation reports was *a*=0.80 (very good) for those familiar with the ontology and *a*= 0.58 (acceptable) for those unfamiliar with it.

**Conclusion:** The ontology can be used for both annotating and writing behaviour change intervention evaluation reports in a consistent and coherent manner, thereby improving evidence comparison, synthesis, replication, and implementation of effective interventions.

## Introduction

Patterns of human behaviour contribute significantly to the global disease burden, as well as to a wide range of environmental and social problems (e.g. Gakidou *et al.*, 2017; Watts *et al.*, 2017). The development of behaviour change interventions, defined as coordinated sets of activities designed to change specified behaviour patterns ( Michie *et al.*, 2011), can be an effective and cost-effective solution to such global problems. Research investigating the development, evaluation and implementation of behaviour change interventions, as well as evidence syntheses, demonstrate striking variability in effectiveness across different studies (see Cochrane database, e.g. Flodgren *et al.*, 2017; Ussher *et al.*, 2012). Understanding this variability is difficult given the complexity of interventions, with variations in content and delivery potentially interacting with each other and with the intervention setting, population and target behaviour.

Being able to specify intervention characteristics in a way that facilitates replication and evidence synthesis is an important step in building evidence efficiently and cumulatively. This requires conceptual frameworks that organise knowledge using clear, coherent, and shared terminology ( Michie *et al.*, 2017). Such frameworks promote communication and collaboration across disciplines and research groups, and can be helpful in advancing knowledge generation to inform intervention development, implementation, evaluation, and reporting ( Craig *et al.*, 2008; Hoffmann *et al.*, 2014; Moher *et al.*, 2001). Another benefit of using conceptual frameworks is that they can enhance researchers’ ability to examine associations between specific intervention components and outcomes ( Sheeran *et al.*, 2017). This allows for a more thorough understanding of interventions and how they bring about their effects which, in turn, can inform the development of more effective interventions.

Previously published classification systems for describing behaviour change interventions include the widely used Behaviour Change Techniques Taxonomy v1 (BCTTv1 ( Michie *et al.*, 2013)), covering intervention content (e.g. Newbury-Birch *et al.*, 2014; Zebis *et al.*, 2016). The BCTTv1 is a hierarchical taxonomy used to classify the potentially ‘active ingredients’ of behaviour change interventions, known as behaviour change techniques (BCTs) ( Michie *et al.*, 2019a; Michie *et al.*, 2013; Michie *et al.*, 2015). BCTTv1, which includes 93 discrete BCTs, has been used to identify and define BCTs in intervention research ( Newbury-Birch *et al.*, 2014; Paul *et al.*, 2017; Young *et al.*, 2014) and to categorise intervention content in evidence syntheses ( Arnott *et al.*, 2014; Jones *et al.*, 2014). By providing a common language with which to describe interventions, BCTTv1 has facilitated a level of rigour and specificity in reporting intervention content that was not previously commonplace ( Sheeran *et al.*, 2017). While BCTTv1 and other classification systems for intervention content (e.g. Hollands *et al.*, 2017) have provided a shared language for specifying intervention content, there are other crucial aspects of behaviour change interventions that have received comparatively little attention, including *how* such content is delivered ( Dombrowski *et al.*, 2016).

### Ontologies

BCTTv1 is an example of a taxonomy, a knowledge representation structure in which a controlled vocabulary of agreed-upon terms is arranged hierarchically. An ***ontology*** is a more expressive structure for organising knowledge (see glossary of italicised terms, Table 1). It includes a controlled vocabulary, unambiguous identifiers for each entity, and additional information such as synonyms and examples of usage. It includes relationships between entities, usually beyond the hierarchical class-subclass relationship as well as a formal, logic-based encoding of domain knowledge where possible ( Arp *et al.*, 2015; Hastings, 2017; Larsen *et al.*, 2017; Michie & Johnston, 2017; Norris *et al.*, 2019). Ontologies enable entities to be compared and integrated across fields of study and allow large datasets to be synthesised efficiently using computational tools (e.g. in biology, the Gene Ontology ( Ashburner *et al.*, 2000).

**Table 1.** Glossary.

Term	Definition	Source
**Annotation**	Process of coding selected parts of documents or other resources to identify the presence of ontology entities.	Michie *et al*., 2018.
**Annotation** **guidance manual**	Written guidance on how to identify and tag pieces of text from intervention evaluation reports with specific codes relating to entities in the ontology.	
**Basic Formal** **Ontology (BFO)**	An upper level ontology consisting of continuants and occurrents developed to support integration, especially of data obtained through scientific research.	Arp *et al*., 2015.
**Entity**	Anything that exists, that can be a continuant or an occurrent as defined in the Basic Formal Ontology.	Arp *et al*., 2015.
**EPPI-Reviewer**	A web-based software program for managing and analysing data in all types of systematic review (meta- analysis, framework synthesis, thematic synthesis etc). It manages references, stores PDF files and facilitates qualitative and quantitative analyses such as meta- analysis and thematic synthesis. It also has a facility to annotate published papers.	Thomas *et al.*, 2010; *EPPI-Reviewer 4:* http://eppi.ioe.ac.uk/eppireviewer4/ *EPPI-Reviewer Web Version:* https://eppi.ioe.ac.uk/ eppireviewer-web/
**GitHub**	A web-based platform used as a repository for sharing code, allowing version control.	https://github.com/
**Inter-rater** **reliability**	Statistical assessment of similarity and dissimilarity of coding between two or more coders. If inter-rater reliability is high this suggests that ontology entity definitions and labels are being interpreted similarly by the coders.	Gwet, 2014. *Handbook of inter-rater reliability: The* *definitive guide to measuring the extent of agreement* *among raters*. Gaithersburg, Advanced Analytics.
**Interoperability**	Two systems are interoperable if data coming from each system can be used by the other system. Note: An ontology is interoperable with another ontology if it can be used together with or re-uses parts from the other ontology	http://www.obofoundry.org/principles/fp-010- collaboration.html
**Issue tracker**	An online log for problems identified by users accessing and using an ontology.	BCIO Issue Tracker: https://github.com/ HumanBehaviourChangeProject/ontologies/issues
**OBO Foundry**	The Open Biological and Biomedical Ontology (OBO) Foundry is a collective of ontology developers that are committed to collaboration and adherence to shared principles. The mission of the OBO Foundry is to develop a family of interoperable ontologies that are both logically well-formed and scientifically accurate.	Smith *et al*., 2007; www.obofoundry.org/
**Ontology**	A standardised framework providing a set of terms that can be used for the consistent annotation (or “tagging”) of data and information across disciplinary and research community boundaries.	Arp *et al*., 2015.
**Parent class**	A class within an ontology that is hierarchically related to one or more child (subsumed) classes such that all members of the child class are also members of the parent class and all properties of the parent class are also properties of the child class.	Arp *et al*., 2015.
**Reconciliation**	The process of discussing differences between the annotations of two paired annotators on the same papers. Differences are discussed before a final reconciled version of coding for each paper is produced.	Stan *et al*., 2014.
**ROBOT**	An automated command line tool for ontology workflows.	Jackson *et al*., 2019, http://robot.obolibrary.org
**Unique resource** **identifier (URI)**	A string of characters that unambiguously identifies an ontology or an individual entity within an ontology. Having URI identifiers is one of the OBO Foundry principles.	http://www.obofoundry.org/principles/fp-003-uris.html
**Web Ontology** **Language (OWL)**	A formal language for describing ontologies. It provides methods to model classes of “things”, how they relate to each other and the properties they have. OWL is designed to be interpreted by computer programs and is extensively used in the Semantic Web where rich knowledge about web documents and the relationships between them are represented using OWL syntax.	https://www.w3.org/TR/owl2-quick-reference/

The potential for ontologies to facilitate knowledge synthesis in behaviour change is being developed in the Human Behaviour-Change Project ( Michie *et al.*, 2018; Michie *et al.*, 2020a; Michie *et al.*, 2020b). This collaboration between behavioural scientists, computer scientists and systems architects is building a database and platform for researchers, practitioners and policy-makers to address variants of the ‘big question’ of behaviour change: “What works, compared with what, how well, with what exposure, with what behaviours (for how long), for whom, in what settings and why?” Answering this involves extending previous work to classify all entities of behaviour change interventions and the relationships between them, i.e. a *Behaviour change intervention ontology* (BCIO), specified by a controlled vocabulary that by the upper level of the BCIO ( Michie *et al.*, 2020b) contains 42 entities. The *Behaviour change intervention delivery* entity of the ontology (i.e. the means by which BCI content is provided), comprises (a) *BCI Source (*i.e., a role played by a person, population or organisation that provides a behaviour change intervention), (b) *BCI Schedule of delivery* (an attribute of a behaviour change intervention that involves its temporal organisation), (c) *BCI Style of delivery* (an attribute of a BCI delivery that encompasses the characteristics of how a behaviour change intervention is communicated), and (d) *BCI Mode of delivery* (an attribute of a BCI delivery that is the physical or informational medium through which a behaviour change intervention is provided).

### Delivery of Behaviour Change Interventions

An important characteristic of behaviour change interventions is the method or methods by which the content (e.g. BCTs) is brought to its target population (i.e. the intervention’s mode of delivery; MoD). MoDs can act synergistically or antagonistically with the intervention techniques in influencing intervention outcomes and effects. An example of this is a meta-analysis of evidence about the effectiveness of smoking cessation interventions, which found effectiveness to be higher with increasing numbers of intervention techniques but only if delivered in person and not when delivered in written form ( Black *et al.*, 2020).

Several systematic reviews have extracted information about MoDs ( Bock *et al.*, 2014; van Genugten *et al.*, 2016; MacDonald *et al.*, 2016), and an annotation scheme for MoD within internet-based interventions has been developed ( Webb *et al.*, 2010). However, MoD has received comparatively little attention in intervention research ( Dombrowski *et al.*, 2016), and there is a lack of clarity and consensus across behavioural intervention research regarding how MoD is defined, what it includes, and how it should be reported. This is in contrast to the reporting of BCTs as the content of behaviour change interventions, for which there is now wide shared understanding, for example, featuring in the Encyclopaedia of Behavioural Medicine ( Michie *et al.*, 2019a) and in many hundreds of publications. The various conceptualisations of MoD, and the lack of a shared language or framework with which to describe it has made the study of interactions between it and other intervention entities difficult to analyse systematically ( Dombrowski *et al.*, 2016). Here, we define MoD as the attribute of BCI delivery that is the informational or physical medium through which a behaviour change intervention is provided ( Michie *et al.*, 2020b). For example, providing someone with information about the health consequences of performing a particular behaviour could be conducted face-to-face (e.g. by a GP), through a poster or leaflet, or through a digital device (e.g. an app). ‘Item 6: How’ of the TIDieR framework highlights the need for researchers to clearly specify the MoD of the intervention. An example of a classification that briefly addresses MoD is the Effective Practice and Organisation of Care (EPOC) Taxonomy ( Effective Practice and Organisation of Care (EPOC), 2015) which includes a category for “How and when care is delivered” and “Information and Communication Technology” including some elements of MoD such as group vs individual care. EPOC was developed specifically to classify delivery of health systems interventions. A framework that systematically describes a vast range of MoD entities that can be implemented in behaviour change interventions for any domain of human behaviour was needed. An ontology provides a mechanism for doing this. The development of an MoD ontology that can be linked to other ontologies relevant to behaviour change interventions would advance scientific understanding, the development and evaluation of interventions and methods for evidence synthesis.

### Aim

The aim of the MoD Ontology is to provide a clear, usable and reliable classification system to specify the MoDs of behaviour change interventions, including single BCTs. The development of an ontology with clear and unambiguously defined terms enables precision of reporting, which in turn promotes evidence synthesis, replication and analyses of associations between MoDs, other intervention characteristics and intervention outcomes.

## Methods

The ontology was developed in seven iterative steps (detailed below), involving reviewing existing classification systems, ***annotation*** of behaviour change intervention reports (including testing of ***inter-rater reliability***) and feedback from international expert stakeholders (outlined in Table 2).

**Table 2.** Steps for developing the Mode of Delivery Ontology.

Phase	Step	Methods
Initial development	1. Developing and piloting a preliminary ontology	Data extraction from 120 BCI reports; - 20 reports for initial draft + 100 for improvements and inter-rater reliability calculations; Group discussions
	2. Requesting feedback on preliminary ontology from expert stakeholders	Open peer review from 25 experts; Group discussions
Testing and refinement	3. Testing & refining ontology through second round of data annotations	Data annotations from 55 BCI reports; inter-rater reliability calculations; inter-rater reliability; Group discussions
	4. Requesting feedback on refined ontology from experts	Open peer-review from 16 experts; consultation with an ontology expert; Group discussions
Consolidation of changes and agreement on final version	5. Testing & finalising ontology through final round of data annotations	Data annotations from 150 BCI reports; inter-rater reliability calculations
	6. Specifying the relations ships between entities	Group discussions
	7. Transforming into machine-readable format	Ontology content was transformed automatically into an OWL ontology using the ROBOT library’s template functionality

MoD, mode of delivery; BCI, Behaviour change intervention.

### Step 1: Development of the preliminary ontology and piloting

Initial descriptions of MoD ***entities*** were extracted from 20 published behaviour change intervention evaluation reports, randomly selected using a random number generator from a larger database of reports annotated by behaviour change techniques and mechanisms of action ( Michie *et al.*, 2018), covering a range of health behaviours. Next, two researchers independently piloted the preliminary MoD ontology with another set of intervention reports, taken from the same database and using the same selection method. Guidance on how to annotate papers for MoD was developed by the research team, providing clear instructions on how to code each entity, including definitions and examples for each. Reports were annotated in batches of 10 until a satisfactory and stable criterion of inter-rater reliability was achieved. Inter-rater reliability of the extent to which researchers capture the same information from a report was measured in two ways. The first was percentage agreement of instances where both researchers had annotated an MoD. The second was the proportion of times annotators agreed on a code when both of them captured the same information from a report. This was calculated at every level of the hierarchy, and it was performed using Cohen’s Kappa ( Cohen, 1960), in Microsoft Excel 365. Kappa values >.61 were deemed as ‘substantial’ and values >.81 as ‘strong’ ( Landis & Koch, 1977). The preliminary ontology was revised and updated iteratively throughout the annotation process. Where there were discrepancies between the two annotators, these were discussed, and amendments were made to the ontology if both annotators judged that these changes would improve clarity. In the case of disagreement, a senior member of the research team was consulted.

### Step 2: Stakeholder review (Round 1)

Nine international behavioural scientists with experience in behaviour change interventions, across a range of behavioural domains, were invited to provide feedback on the structure, content and terminology of the preliminary MoD Ontology. Following small adjustments based on this feedback, the MoD Ontology was published online, and a wider international research community was invited through mailing lists to submit feedback using an open *Qualtrics* form presenting the preliminary MoD structure, and entity labels and definitions (see https://osf.io/eyn3b/ ( West *et al.*, 2020)). Twenty-five behavioural scientists responded to indicate whether 1) there were any entities missing, 2) the structure was coherent, 3) there were changes needed in the terminology of the labels and definitions, and 4) there were additional suggestions for improvement.

### Step 3: Inter-rater reliability testing (Round 2)

The revised version was used to annotate MoD entities in a set of 55 published reports, randomly selected using a random number generator from the database mentioned in Step 1 ( Michie *et al.*, 2018). These papers covered the behavioural domains of physical activity, diet and smoking. Annotation of the reports was conducted independently by two researchers. The annotation process was carried out in batches of five papers. After every batch, annotations were compared, and discrepancies discussed. Inter-rater reliability was calculated using the same procedure as in Step 1. Where there were discrepancies, consensus was reached through discussion.

### Step 4: Stakeholder review (Round 2)

Experts who provided feedback in Step 2 were invited to submit feedback on the revised ontology. Experts were sent an email with a request to review the structure, labels and definitions of each entity, and indicate whether the structure was coherent and whether there was anything missing and provide suggestions for improved terminology. During this step, an ontology expert (JH) was consulted regarding the structure and definitions.

### Step 5: Inter-rater reliability testing (Round 3)

To test the range of applicability of this revised version of the MoD Ontology (as well as the ***annotation guidance manual***), we conducted a final round of annotations as part of the annotations being conducted in the Human Behaviour-Change Project. First, two developers of the MoD ontology annotated reports that were selected from a database of reports used in the Human Behaviour-Change Project ( Michie *et al.*, 2017) (see https://osf.io/myje6/ ( West *et al.*, 2020)). These annotations were conducted using ***EPPI reviewer 4*** software ( Thomas *et al*., 2010). An open alternative to this software used for annotation is PDFAnno ( Shindo *et al.*, 2018). All reports were randomised controlled trials from one of three datasets: Cochrane Reviews, papers annotated for behaviour change techniques and papers from the IC-SMOKE project ( De Bruin *et al.*, 2016) (list of systematic reviews included as *Extended data* at https://osf.io/myje6/ ( West *et al.*, 2020)). There was a reconciliation process after the first batch of 10, followed by any necessary amendments to the annotation manual. These amendments mainly involved the inclusion of examples (e.g. illustrating when to code or not to code certain pieces of information as MoD).

To examine the usability of the MoD Ontology for researchers and intervention developers with no prior knowledge of the MoD Ontology, we conducted a final round of inter-rater reliability assessment by asking two researchers unfamiliar with the ontology and without specific expertise in modes of delivery to annotate a random sample of randomised controlled trials from a database of papers annotated by BCTs, with no restrictions on the outcome behaviour. Inter-rater reliability was assessed using Krippendorff’s Alpha ( Hayes & Krippendorff, 2007), using Python 3.6 (code available on GitHub ( Finnerty & Moore, 2020)).

### Step 6: Specifying relationships within the MoD Ontology

The research team developed relationships between ontology entities to formally capture the types of knowledge that are present in the ontology. The relationships were specified following best practices from Basic Formal Ontology (BFO) described in Arp *et al.* (2015) and Relation Ontology ( Smith *et al.*, 2005). Relationships can be generic and shared across multiple ontologies (e.g the “is a” relationship between classes where one class is a subclass of another class, or the “part of” relationship which captures the relationship between wholes and their parts) or they can be domain specific, which are introduced when needed to formally capture relationships unique to a given domain.

### Step 7: Making the MoD Ontology machine-readable and available online

The MoD Ontology was initially developed as a table of entities, with separate rows for each entity annotated in columns for different types of annotation, including a primary label, definition, synonyms and relationships. When the MoD Ontology was at a stable level of development for initial release, it was converted into the ***Web Ontology Language*** **(OWL)**( Antoniou & van Harmelen, 2004) format, enabling it to be viewed and visualised using ontology software such as Protégé and to be compatible with other ontologies and software tools. The conversion to OWL used the ***ROBOT*** ontology toolkit library ( Jackson *et al.*, 2019), which provides a facility to create well-formatted ontologies from templates. A ROBOT template can be prepared easily in common spreadsheet software, annotated with instructions for translation from spreadsheet columns to OWL language and metadata entities. Within the input template spreadsheet, separate columns represent the entity ID (e.g. BCIO:011004), name, definition, relationship with other entities, examples and synonyms.

This OWL version of the MoD Ontology was then stored on the project GitHub repository ( Finnerty & Moore, 2020), as ***GitHub*** has an ***issue tracker***, which allows feedback to be submitted by members of the community that can be responded to, and if necessary, addressed in subsequent releases. When the full BCIO has been finalised, it will be submitted to the ***OBO Foundry*** ( Smith *et al.*, 2007).

## Results

### Step 1: Development of the preliminary ontology and piloting

The data extracted from the behaviour change intervention reports led to the identification of 160 unique entities, which were represented in a four-level hierarchical structure, as well as two ‘cross-cutting’ entities (a description of the preliminary version is available as *Extended data* at https://osf.io/gu5ke/ ( West *et al.*, 2020)). A hundred reports were annotated, with adjustments made to the ontology as a result of the first 70; the ontology was stable for the final 30 reports. Average agreement between annotators for each batch of 10 reports varied between 72% and 95%. Inter-rater reliability was calculated for each level of the hierarchy separately and considered to be ‘good’ for all levels (% agreement 86.6 to 97.8; Kappa 0.68 to 0.97). Reliability was also calculated for each of the cross-cutting entities (Kappa = .55 and .75). Further details on the inter-rater reliability and changes made to the MoD Ontology in this step can be found as *Extended data* at: https://osf.io/r3wn2/ ( West *et al.*, 2020).

### Step 2: Stakeholder review (Round 1)

Feedback on the MoD ontology through the open review feedback form was received by 25 experts, of which 18 were from universities, 5 were from commercial sector organisations, 1 from public sector organisations and 1 from third sector. Twelve experts were from the United Kingdom, 2 from the United States of America, 3 from Ireland, 1 from Canada, 1 from the Netherlands, 1 from New Zealand, and we have no information about the country for the remaining 5 experts. These data were collated, synthesised, and discussed among the research team. This led to further amendments to the structure, content and terminology (full details on the feedback and corresponding changes made to the MoD Ontology are available as *Extended data* at https://osf.io/95n3a/ ( West *et al.*, 2020)).

### Step 3: Inter-rater reliability testing (Round 2)

For the 55 papers annotated in this round, agreement for whether a particular entity was considered an MoD was 61%; and agreement on the specific MoD code assigned was 87.9% (Kappa = .857) (inter-rater reliability results are available as *Extended data* at https://osf.io/sw2jv/ ( West *et al.*, 2020)).

### Step 4: Stakeholder review (Round 2)

Feedback was received from 16 of the 25 experts invited. Based on this, the following changes were made: 1) the entities “other” and “unclear” were removed, as all entities represented in an ontology need to be fully specified; and (2) increased clarity was provided on how the cross-cutting entities related to the other upper-level classes (see https://osf.io/3zhbc/ ( West *et al.*, 2020) for more details").

For the revised version, definitions were developed using pre-specified guidance, with the standard format of definitions being: A is a B that C, or involves or relates to C in some way, where A is the class being defined, B is a ***parent class*** and C describes a set of properties of A that distinguish it from other members of B ( Michie *et al.*, 2019b).

### Step 5: Inter-rater reliability testing (Round 3)

For the annotations conducted by researchers familiar with the MoD ontology, a very good agreement ( *a*=0.80) was achieved after annotating 50 reports (25 smoking and 25 physical activity). For the annotations conducted by researchers unfamiliar with the ontology, acceptable agreement ( *a*=0.58) was achieved after annotating 96 papers, targeting various behaviours (26 physical activity; 22 diet; 13 alcohol; 11 treatment adherence; nine sexual behaviours; seven multiple health behaviours; two for prescription, smoking, and screening, respectively; and one paper for organ donation and one for oral health) ( Hayes & Krippendorff, 2007) (inter-rater reliability results are available as Extended data at https://osf.io/efp4x/ ( West *et al.*, 2020)).

### Step 6: Specifying relationships within the MoD Ontology

Currently, the only relationship used in the ontology represent its hierarchical structure, i.e. “subclass of” (is_a) relationships (e.g. *face to face MoD* “is_a” *human interactional MoD*). Formal representations of knowledge using explicit logical relationships allow computational tools to perform additional checks and inferences to enhance the resulting consistency of reporting for complex interventions.

### Step 7 - Making the MoD Ontology machine-readable and available online

A downloadable version of the final MoD Ontology can be found on GitHub ( Finnerty & Moore, 2020). The hierarchical structure, labels, ***uniform resource identifiers (URIs)*** and definitions for all entities are described in Table 3. The ontology is accompanied by an annotation manual that provides guidance on how to annotate for these entities in reports of behaviour change interventions (available as *Extended data* at https://osf.io/4j2xh/ ( West *et al.*, 2020)). The final MoD Ontology presents a four-level hierarchical structure comprising 65 entities. There are 15 upper-level classes: 1.1. Informational MoD, 1.2. Environmental change MoD; 1.3. Somatic MoD; 1.4. Somatic alteration MoD; 1.5. Individual-based MoD vs 1.6. Pair-based MoD, vs 1.7. Group-based MoD; 1.8. Uni-directional MoD vs. 1.9. Interactional MoD; 1.10. Synchronous MoD vs. 1.11. Asynchronous MoD; 1.12. Push MoD vs. 1.13. Pull MoD; 1.14. Gamification MoD; 1.15. Arts feature MoD. The first upper-level classes include lower level entities (sub-classes). For example, *Informational MoD* includes *Printed material MoD*, which includes sub-classes of *Letter MoD*, *Public notice MoD*, *Printed publication MoD*, and *Labelling MoD*. Entities from 1.5 to 1.15 correspond to entities that can be present at the same time as at least one of the other MoD. For example, an intervention that is *delivered through face to face* (sub-class of *Human interactional MoD*), can also be classified as an *Individual-based* or *Group-based MoD.* It is worth noting that, given the exponential growth in new technologies, this MoD Ontology captures a specific moment in time, and will need updating as technologies and methods develop.

**Table 3.** Entity labels, definitions, URIs and examples of usage for Mode of Delivery Ontology entities.

Upper-Level	Sub-Level 1	Sub-Level 2	Sub-Level 3	Definition	Examples of usage
Informational mode of delivery BCIO:011001				Mode of delivery that involves intentional transmission of a representation of the world to an intervention recipient with the aim of changing that person's representation of the world.	This includes delivery of rewards, prompts, and cues that result in learning and information about the environment and environmental contingencies.
	Human interactional mode of delivery BCIO:011002			Informational mode of delivery that involves a person as intervention source who interacts with an intervention recipient in real time.	
		Face to face mode of delivery BCIO:011003		Human interactional mode of delivery that involves an intervention source and recipient being together in the same location and communicating directly.	
		At-a-distance mode of delivery BCIO:011004		Human interactional mode of delivery that involves an intervention source and recipient being in different locations and communicating through a communication channel.	
	Printed material mode of delivery BCIO:011005			Informational mode of delivery that involves use of printed material.	Can include paper, acetate, text, diagrams and photographic images.
		Letter mode of delivery BCIO:011006		Printed material mode of delivery that involves a letter or postcard that can be sent through the post or handed directly to the recipient.	
		Public notice mode of delivery BCIO:011007		Printed material mode of delivery that involves display of a poster, sign or notice in a public location.	
		Printed publication mode of delivery BCIO:011008		Printed material mode of delivery that involves use of a printed publication.	Includes leaflets, brochures, newspapers, newsletter, booklets, magazines, manuals or worksheets.
		Labelling mode of delivery BCIO:011009		Printed material mode of delivery that involves information printed on a product or its packaging, or a label attached to or included with, a product or its packaging, and aims to convey information about that product.	
	Electronic mode of delivery BCIO:011010			Informational mode of delivery that involves electronic technology in the presentation of information to an intervention recipient.	
		Television mode of delivery BCIO:011011		Electronic mode of delivery that involves presentation of information that is broadcast and displayed by television.	Includes internet and satellite television.
		Mobile digital device mode of delivery BCIO:011012		Electronic mode of delivery that involves presentation of information by a handheld mobile digital device that can store, retrieve and process data.	
		Computer mode of delivery BCIO:011013		Electronic mode of delivery that involves presentation of information by a desktop or laptop computer.	
		Electronic billboard mode of delivery BCIO:011014		Electronic mode of delivery that involves presentation of information by an electronic screen positioned in a public location.	
		Wearable electronic device mode of delivery BCIO:011015		Electronic mode of delivery that involves presentation of information by an electronic screen positioned in a public location.	Includes a watch, clip-on device, spectacles, in-ear devfice, vibrating device.
		Electronic environmental object mode of delivery BCIO:011016		Electronic mode of delivery that involves an electronic device positioned in the environment of the intervention recipient that can gather information and respond to commands.	Includes robots, and 'internet of things'.
		3-D projection mode of delivery BCIO:011017		Electronic mode of delivery that involves presentation of a 3-D image.	Includes hologram but does not include virtual reality headsets.
		Virtual reality mode of delivery BCIO:011018		Electronic mode of delivery that involves use of virtual reality through a virtual reality headset and optionally body movement sensors.	
		Playable electronic storage mode of delivery BCIO:011019		Electronic mode of delivery that involves presentation of information stored on an object that is inserted into a playing device.	Includes cassettes, video tapes, DVDs, CDs.
		Radio broadcast mode of delivery BCIO:011020		Electronic mode of delivery that involves presentation of audio information that is broadcast and received by a radio receiver.	Includes cassettes, video tapes, DVDs, CDs.
		Call mode of delivery BCIO:011021		Electronic mode of delivery that involves a communication process in which a signal is sent by a caller to a recipient to alert them of the communication intent, giving the recipient the opportunity to engage with the communication.	Includes automated calls and audio messaging.
			Audio call mode of delivery BCIO:011022	Call mode of delivery that involves only audio information in the communication.	
			Video call mode of delivery BCIO:011023	Call mode of delivery that involves video and audio information in the communication.	
			Messaging mode of delivery BCIO:011024	Call mode of delivery that involves textual information in the communication.	Text message can include emojis, and additional audio and pictorial material. Includes SMS, WhatsApp and other messaging services.
		Email mode of delivery BCIO:011025		Electronic mode of delivery that involves communication by email.	
		Video game mode of delivery BCIO:011026		Electronic mode of delivery that involves the intervention recipient playing a computer game	
		Website mode of delivery BCIO:011027		Electronic mode of delivery that involves the intervention recipient interacting with a website.	
		Mobile application mode of delivery BCIO:011028		Electronic mode of delivery that involves the intervention recipient interacting with a mobile application.	
		E-book mode of delivery BCIO:011029		Electronic mode of delivery that involves the intervention recipient being given access to an e-book.	
	Audio informational mode of delivery BCIO:011030			Informational mode of delivery that involves sound.	
	Visual informational mode of delivery BCIO:011031			Informational mode of delivery that involves visual images.	
	Textual mode of delivery BCIO:011032			Informational mode of delivery that involves written text.	
Environmental change mode of delivery BCIO:011033				Mode of delivery that involves changing the physical shape, size, structure or appearance of objects in the environment of the intervention recipient.	This does not include use of textual or pictorial information. It includes lighting, speed humps, use of music, shape and size of containers of consumables.
Somatic mode of delivery BCIO:011034				Mode of delivery that involves devices or substances that alter bodily processes or structure.	
	Ingestion mode of delivery BCIO:011035			Somatic mode of delivery that involves ingestion of a chemical into the body.	
		Transdermal mode of delivery BCIO:011036		Ingestion mode of delivery that involves ingestion of a chemical through the skin.	
		Alimentary mode of delivery BCIO:011037		Somatic mode of delivery that involves ingestion of a chemical through the stomach or intestine.	
			Pill mode of delivery BCIO:011038	Alimentary mode of delivery that involves swallowing of a pill or oral capsule.	
			Ingestible liquid mode of delivery BCIO:011039	Alimentary mode of delivery that involves swallowing of a liquid.	
		Buccal mode of delivery BCIO:011040		Ingestion mode of delivery that involves absorption of a chemical through the lining of the buccal cavity.	
		Inhalation mode of delivery BCIO:011041		Ingestion mode of delivery that involves absorption of a chemical through the upper airways or lungs by inspiration.	
		Injection mode of delivery BCIO:011042		Ingestion mode of delivery that involves a chemical being introduced into body tissue through a hollow needle that punctures the skin.	
			Subcutaneous injection mode of delivery BCIO:011043	Injection mode of delivery in which the tissue receiving the chemical is subcutaneous tissue.	
			Intravenous injection mode of delivery BCIO:011044	Injection mode of delivery in which the tissue receiving the chemical is subcutaneous tissue.	
			Intramuscular injection mode of delivery BCIO:011045	Injection mode of delivery in which the tissue receiving the chemical is muscle.	
		Wearable ingestion mode of delivery BCIO:011046		Injection mode of delivery in which the tissue receiving the chemical is muscle.	Includes insulin pump.
	Chewable substance mode of delivery BCIO:011047			Ingestion mode of delivery that involves chewing of a soft material.	This includes chewing gum. Often involves ingestion of a chemical that is released by chewing and absorbed through the lining of the buccal cavity.
	Physical stimulus mode of delivery BCIO:011048			A mode of delivery that involves application of a physical stimulus to the body.	
		Light exposure mode of delivery BCIO:011049		Physical stimulus mode of delivery that involves exposure of light to the body.	
		Temperature mode of delivery BCIO:011050		Physical stimulus mode of delivery that involves application of heat or cold to the body.	
		Electrical stimulation mode of delivery BCIO:011051		Physical stimulus mode of delivery that involves application of electrical stimulation to the body.	
		Physical pressure mode of delivery BCIO:011052		Physical stimulus mode of delivery that involves application of physical pressure to the outside of the body.	Includes massage.
		Wearable stimulus mode of delivery BCIO:011053		Physical stimulus mode of delivery that involves a device that is worn on the body.	
Somatic alteration mode of delivery BCIO:011054				Mode of delivery that involves modifying the structure of the body of the recipient of the intervention.	Includes surgery.
Individual-based mode of delivery *BCIO:011055				Mode of delivery that involves one recipient in the location where the intervention is delivered.	
Pair-based mode of delivery *BCIO:011056				Mode of delivery that involves two recipients in the location where the intervention is delivered who have an interpersonal relationship.	
Group-based mode of delivery *BCIO:011057				Mode of delivery that involves three or more people in the location where the intervention is delivered.	
Uni-directional mode of delivery **BCIO:011058				Mode of delivery in which the only causal influence is from the intervention source to the recipient.	
Interactional mode of delivery **BCIO:011059				Mode of delivery in which there is causal influence from the intervention source to the recipient and from the recipient to the source.	
Synchronous mode of delivery ***BCIO:011060				Mode of delivery that involves delivery and receipt of the intervention or its components occurring at the same time or very close in time.	
Asynchronous mode of delivery ***BCIO:011061				Mode of delivery that involves receipt of the intervention or its components taking place a significant period of time after delivery.	
Push mode of delivery ****BCIO:011062				Mode of delivery that is not dependent on actions on the part of the intervention recipient.	
Pull mode of delivery ****BCIO:011063				Mode of delivery that requires some action on the part of the recipient.	
Gamification mode of delivery BCIO:011064				Mode of delivery that involves application of typical elements of game playing to other areas of activity, typically as an online marketing technique to encourage engagement with a product or service.	Includes point scoring, competition with others, and rules of play.
Arts feature mode of delivery BCIO:011065				Mode of delivery that involves application of creativity on the part of the intervention recipient.	Includes art therapy, music therapy, dance and acting.

**Note.** Entity IDs correspond to Behaviour Change Intervention Ontology (BCIO);* Only one of individual-based, group-based or pair-based mode of delivery will apply; **only one of uni-directional or interactional mode of delivery will apply; ***only one of synchronous or asynchronous mode of delivery will apply; **** only one of push or pull mode of delivery will apply.

## Discussion

Given the lack of classification systems providing comprehensive coverage of how behaviour change interventions and techniques are delivered, we developed the first ontology of modes of delivery (MoD). This ontology consists of 65 entities organised in 15 upper-level entities. Inter-rater reliability was found to be 0.80 (very good) for those familiar with the ontology and 0.58 (acceptable) for those unfamiliar with it, as assessed by Krippendorff’s alpha. Together with *Source, Schedule* and *Style* it represents the characteristics of *Delivery* of a behaviour change intervention. Ontologies aim to be dynamic representations that are updated according to new evidence on entities and relationships. As with other lower level ontologies that form part of the BCIO ( Michie *et al.*, 2020b), the MoD Ontology will be improved upon and refined through application and feedback by users.

The MoD Ontology contributes to the growing number of methodological resources now freely available to intervention researchers (e.g. Bartholomew *et al.*, 2011; Hoffmann *et al.*, 2014; Hollands *et al.*, 2017; Michie *et al.*, 2013). For example, a Theory and Techniques Tool available for free online, provides an interactive dataset of links between BCTs and their mechanisms of action (i.e. the processes through which BCTs have their effects). The tool was informed by data from evidence synthesis ( Carey *et al.*, 2019) and expert consensus ( Connell *et al.*, 2019), which were triangulated ( Johnston *et al.*, 2018); all three sets of data are available in the tool.

The MoD Ontology contributes to a larger programme of work developing ontologies for other intervention components, the Human Behaviour-Change Project ( Michie *et al.*, 2018; Michie *et al.*, 2020a). Within this project, lower level ontologies are being developed for intervention-related entities of content, delivery, tailoring, context, engagement, mechanism of action, and outcome behaviour within the BCIO ( Michie *et al.*, 2020b). These ontologies have been developed using an explicit, standardised, and tested method for ontology development created within the Human Behaviour-Change Project ( Wright *et al.*, 2020). As the development of the MoD ontology started prior to the development of the BCIO, the process of development was slightly different from the one described in this collection ( Wright *et al.*, 2020), containing more rounds of expert feedback and inter-rater reliability testing.

The MoD ontology provides a crucial contribution to the much needed body of research examining the links between MoDs and the content of behaviour change interventions, using the BCTTv1 or other classification systems of techniques (e.g. Knittle *et al.*, 2020; Kok *et al.*, 2016). For example, coding existing behaviour change interventions for their modes of delivery and BCTs can increase our understanding of which mode(s) of delivery are the most effective in delivering a given BCT. Further, by linking with other HBCP ontologies characterising behaviour change interventions, it will be possible to go a step further and identify which MoD(s) are more appropriate for different behaviours, populations, contexts, if they need to be tailored, and their potential for reach and engagement.

### Strengths and limitations

These ontologies provide a framework for applying machine learning and reasoning algorithms to synthesise and interpret evidence, as well as predict outcome. This allows real-time up-to-date evidence to be interrogated by users such as policy-makers, planners and intervention designers to answer variants of the “big question”: “What works, compared with what, how well, with what exposure, with what behaviours (for how long), for whom, in what settings and why?”, across a wide range of contexts. This body of work has the potential to have far-reaching use by and implications for policy-makers, practitioners and researchers - for example, by informing evidence-based guidelines and identifying knowledge gaps.

Further, the use of entity IDs for each entity in the ontology provides a machine-readable identifier for integration in future systems and also allows ***interoperability*** between existing ontologies.

Several limitations should be noted about the development process, and the resulting MoD Ontology. Given the rapid growth in new technologies and the fast-moving pace of behavioural science research, the MoD Ontology will need updating and refining as existing methods develop and new methods emerge. However, this is common to all ontologies and indeed considered ‘best practice’ in ontology development ( Arp *et al.*, 2015). Secondly, the intervention reports included in the annotation process were from two larger projects, the Theory and Techniques Project ( Michie *et al.*, 2018) and the Human Behaviour-Change Project ( Michie *et al.*, 2017). The intervention reports annotated within the ontology development mainly addressed two health-related behaviours, smoking cessation and physical activity; there is always the possibility that other literature within and outside the health domain may indicate modes of delivery not captured in our set of papers or by our group of experts. However, external inter-rater reliability was tested across diverse behaviours and found to be acceptable. Future applications of the ontologies to a wider collection of non-health related behaviours and contexts is likely to extend and improve the ontology. The inter-rater reliability of the annotations conducted by coders unfamiliar with the ontology was lower than that found in other ontologies of the BCIO such as the Intervention Setting Ontology ( Norris *et al.*, 2020), a result that can be explained by the complexity of this ontology. Nonetheless, the coding guidelines were refined throughout the process and the level of reliability increased considerably between the first and second sets of 50 papers. It is our recommendation that anyone interested in using the MoD ontology should first familiarise themselves with the MoD entities (labels, definitions and examples) and their relationships, read the coding manual, and conduct some trial annotations and assessment of reliability.

## Conclusions

The MoD Ontology provides a foundation on which future research can build, and its development is intended to be an ongoing and collaborative process. By providing greater clarity about *how* an intervention and its components are delivered, researchers can add to knowledge as to how MoDs influence intervention effectiveness, both directly and in interaction with other intervention-related entities. This will inform the selection of appropriate MoDs for interventions.

## Ethics

Ethical approval was granted by University College London’s ethics committee (CEHP/2016/555). Participant consent was gained from the first page of the online Qualtrics survey.

## Data availability

### Underlying data

The BCIO is available from: https://github.com/HumanBehaviourChangeProject/ontologies.

Archived ontology as at time of publication: https://doi.org/10.5281/zenodo.3824323 ( Norris *et al.*, 2021).

License **:** CC-BY 4.0.

### Extended data

Open Science Framework: Human Behaviour-Change Project. https://doi.org/10.17605/OSF.IO/UXWDB ( West *et al.*, 2020).

This project contains the following extended data related to this method:

- Copy of feedback form (PDF)
- Papers used in HBCP annotations (PDF)
- Description of the preliminary version of the MoD Ontology (PDF)
- Step 1 - Inter-Rater Reliability of the preliminary version of the Mode of Delivery Ontology (PDF)
- Feedback Report feedback and corresponding changes made to the Ontology (PDF)
- Step 3 - Inter-Rater Reliability of the preliminary version of the Mode of Delivery Ontology (PDF)
- General guidance for Mode of Delivery Ontology (PDF)

Data are available under the terms of the Creative Commons Attribution 4.0 International license (CC-BY 4.0).

Code used to calculate alpha for IRR: https://github.com/HumanBehaviourChangeProject/Automation-InterRater-Reliability.

Archived code as at time of publication: https://doi.org/10.5281/zenodo.3833816 ( Finnerty & Moore, 2020).

License: GNU General Public License v3.0
