# Supplementary material for: Delivering Behaviour Change Interventions: Development of a Mode of Delivery Ontology
Source: Wellcome Open Res. 2021 Feb 26;5:125. Originally published 2020 Jun 10. [Version 2] doi: 10.12688/wellcomeopenres.15906.2 (PMC7993627; doi:10.12688/wellcomeopenres.15906.2)
Supplement: Peer review history

# Peer review: Reviewer response for version 2

**DOI:** 10.21956/wellcomeopenres.18343.r42863

**Published:** 2021-03-23

**Authors:** Lucie Byrne-Davis

**Affiliations:** 1 Division of Medical Education, University of Manchester, Manchester, UK

I have no further comments to make. The authrors have responded fully to each of my initial comments. This is a very interesting paper that provides insight into the development of this much needed ontology.

Is the work clearly and accurately presented and does it cite the current literature?

Partly

If applicable, is the statistical analysis and its interpretation appropriate?

Yes

Are all the source data underlying the results available to ensure full reproducibility?

Yes

Is the study design appropriate and is the work technically sound?

Yes

Are the conclusions drawn adequately supported by the results?

Yes

Are sufficient details of methods and analysis provided to allow replication by others?

Yes

Reviewer Expertise:

Behaviour change in health settings, particularly  health worker practice change; health worker education

I confirm that I have read this submission and believe that I have an appropriate level of expertise to confirm that it is of an acceptable scientific standard.

# Peer review: Reviewer response for version 2

**DOI:** 10.21956/wellcomeopenres.18343.r42862

**Published:** 2021-03-08

**Authors:** Ann DeSmet

**Affiliations:** 1 Université Libre de Bruxelles, Brussels, Belgium

All previous comments were sufficiently addressed.

Is the work clearly and accurately presented and does it cite the current literature?

Yes

If applicable, is the statistical analysis and its interpretation appropriate?

Yes

Are all the source data underlying the results available to ensure full reproducibility?

Yes

Is the study design appropriate and is the work technically sound?

Yes

Are the conclusions drawn adequately supported by the results?

Yes

Are sufficient details of methods and analysis provided to allow replication by others?

Partly

Reviewer Expertise:

health psychology, behaviour change, digital health interventions, serious games

I confirm that I have read this submission and believe that I have an appropriate level of expertise to confirm that it is of an acceptable scientific standard.

# Peer review: Reviewer response for version 1

**DOI:** 10.21956/wellcomeopenres.17447.r39974

**Published:** 2020-09-14

**Authors:** Ann DeSmet

**Affiliations:** 1 Université Libre de Bruxelles, Brussels, Belgium

The authors describe their approach and results in building an ontology of mode of delivery of interventions in this paper. The paper is well-written, clearly structured and methodologically sound. I have listed a few suggestions and minor comments below:

- I personally appreciate the initiative the authors have taken here. Having created and evaluated serious game interventions, I have noticed how certain techniques may be recommended or effective in one type of intervention, but may not work so well when delivered in a game format. I agree that a detailed description of delivery modes and their structure (what belongs to which group) is necessary, but I was wondering if the authors could also provide more detailed suggestions for future use in the discussion/conclusion part.
  For example, the BCTv1 is mentioned in the introduction, but how do the authors plan to make the connection between this taxonomy and the ontology? Could they shed more light on future initiatives to clarify the importance of this work to the reader?
  The authors also refer to several taxonomies of techniques that exist (it may be useful to also refer to the Intervention Mapping protocol list that is relatively well-known - Kok, G., Gottlieb, N. H., Peters, G. J. Y., Mullen, P. D., Parcel, G. S., Ruiter, R. A., ... & Bartholomew, L. K. (2016). A taxonomy of behaviour change methods: an intervention mapping approach. *Health psychology review*, *10*(3), 297-312 ^1^. Could they clarify where this ontology is inherently linked to the taxonomy or could in the future also be used with other BCT taxonomies, as in a type of open platform communication?
- In the method part it was sometimes difficult to see the link between the text and Table 2. The text, for example, mentions 20 pilot reports in step 1, and then another 'set of interventions'. Table 2 then shows 120 BCI reports were extracted. Why 120? How did the authors decide this was an appropriate number? Same for step 3 (55 reports).
  Could more information be provided on the database? Are these reports that maybe already follow a certain protocol of annotation, could this create some bias?
  The authors mention 'mailing lists' as a way to recruit the experts. Could they provide more information on the mailing lists, or characteristics of experts?
- Could the authors elaborate more on the potential reasons for discrepancies in interrater reliability 'whether a particular entity was considered an MoD was 61%; and agreement on the specific MoD code assigned was 87.9%' in round 2?
- Step 5: could it be that the lower agreement between raters was not related to the fact that they were less familiar with the ontology, but by the fact that there were was a wider variety in target behaviors in this selection of reports? Taxonomies are also mostly applied to diet, physical activity, addictive behaviours; could it be that the ontology does not fit as well with screening, infectious diseases etc?
- Table 2 mentions inter-rater reliability twice for step 3: typo?
- Table 3: definition of video game delivery seems to copy-pasted from the level above?
- Table 3: Somatic alteration mode of delivery - also typo (copy-paste above)?

Is the work clearly and accurately presented and does it cite the current literature?

Yes

If applicable, is the statistical analysis and its interpretation appropriate?

Yes

Are all the source data underlying the results available to ensure full reproducibility?

Yes

Is the study design appropriate and is the work technically sound?

Yes

Are the conclusions drawn adequately supported by the results?

Yes

Are sufficient details of methods and analysis provided to allow replication by others?

Partly

Reviewer Expertise:

health psychology, behaviour change, digital health interventions, serious games

I confirm that I have read this submission and believe that I have an appropriate level of expertise to confirm that it is of an acceptable scientific standard.

## References

[1] : A taxonomy of behaviour change methods: an Intervention Mapping approach. *Health Psychol Rev*.2016;10(3) : 10.1080/17437199.2015.1077155 297-312 10.1080/17437199.2015.1077155 26262912PMC4975080

# Response

**Published:** 2021-02-15

**Authors:** Marta Marques

**Affiliations:** Trinity College Dublin, Ireland

**The authors describe their approach and results in building an ontology of mode of delivery of interventions in this paper. The paper is well-written, clearly structured and methodologically sound. I have listed a few suggestions and minor comments below:**

 We appreciate the reviewer’s positive feedback. We have addressed all comments.

**Personally appreciate the initiative the authors have taken here. Having created and evaluated serious game interventions, I have noticed how certain techniques may be recommended or effective in one type of intervention, but may not work so well when delivered in a game format. I agree that a detailed description of delivery modes and their structure (what belongs to which group) is necessary, but I was wondering if the authors could also provide more detailed suggestions for future use in the discussion/conclusion part.**

**For example, the BCTv1 is mentioned in the introduction, but how do the authors plan to make the connection between this taxonomy and the ontology? Could they shed more light on future initiatives to clarify the importance of this work to the reader?**

 Thank you for this important remark. We have added a new paragraph to the discussion as follows: “The MoD ontology provides a crucial contribution to the much needed body of research examining the links between MoDs and the content of behaviour change interventions, using the BCTTv1 or other classification systems of techniques (e.g. Knittle *et al.,* 2020; Kok *et al.,* 2016). For example, coding existing behaviour change interventions for their modes of delivery and BCTs can increase our understanding of which mode(s) of delivery are the most effective in delivering a given BCT.  Further, by linking with other HBCP ontologies characterising different aspects of behaviour change interventions, it will be possible to go a step further and identify which MoD(s) are more appropriate for different behaviours, populations, contexts, if they need to be tailored, and their potential for reach and engagement.”

**Taxonomies of techniques that exist (it may be useful to also refer to the Intervention Mapping protocol list that is relatively well-known - Kok, G., Gottlieb, N. H., Peters, G. J. Y., Mullen, P. D., Parcel, G. S., Ruiter, R. A., ... & Bartholomew, L. K. (2016). A taxonomy of behaviour change methods: an intervention mapping approach. *Health psychology review*, *10*(3), 297-312 ^1^. Could they clarify where this ontology is inherently linked to the taxonomy or could in the future also be used with other BCT taxonomies, as in a type of open platform communication?**

 Thank you for this suggestion. We have added information related to this to the paragraph presented in the previous comment.  

**In the method part it was sometimes difficult to see the link between the text and Table 2. The text, for example, mentions 20 pilot reports in step 1, and then another 'set of interventions'. Table 2 then shows 120 BCI reports were extracted. Why 120? How did the authors decide this was an appropriate number? Same for step 3 (55 reports).**

 Thank you for noticing this. In step 1 there was an initial extraction of 20 reports for the first skeleton of the ontology and then 100 papers more were annotated to improve the coverage and specificity of the ontology and test its reliability. We have amended the table as follows: “Data extraction from 120 BCI reports: 20 reports for initial draft + 100 for improvements and inter-rater

 reliability calculations”. The number of papers was not pre defined, the coders kept reviewing until an adequate Kappa was reached. The same was true for the number of papers in step 3.

**Could more information be provided on the database? Are these reports that maybe already follow a certain protocol of annotation, could this create some bias?**

 Thank you for pointing this out. The 55 reports came from a collection of articles assembled for a previous project in our research group (Michie et al., 2018). These are articles in which authors described links between behaviour change techniques and intervention mechanisms of action.  Mode of delivery might be described in more detail in these papers where other aspects of interventions are also specified in detail. This greater level of nuance is likely to be a greater challenge to create ontology categories to fit, and so make achieving good inter-rater reliability more difficult.

**The authors mention 'mailing lists' as a way to recruit the experts. Could they provide more information on the mailing lists, or characteristics of experts?**

 We thank the reviewer for this important point. Invitations to potential participants were sent out via third-party mailing lists (e.g. conference). We have some data on the characteristics of the experts who participated, such as the type of organisations reviewers were from and countries. We also have a list of the specific institutions they were from.

 We have added this information to the results section, step 2 as follows: “Feedback on the MoD ontology through the open review feedback form was received by 25 experts, of which 18 were from universities, 5 were from commercial sector organisations, 1 from public sector organisations and 1 from third sector. Twelve experts were from the United Kingdom, 2 from the United States of America, 3 from Ireland, 1 from Canada, 1 from the Netherlands, 1 from New Zealand, and we have no information about the country for the remaining 5 experts.”

**Could the authors elaborate more on the potential reasons for discrepancies in interrater reliability 'whether a particular entity was considered an MoD was 61%; and agreement on the specific MoD code assigned was 87.9%' in round 2?**

 The first element corresponds to recognizing that part of the text contains a description of a mode of delivery. One of the reasons for this lower agreement can be due to the fact that many papers describe mode of delivery poorly and it is stated in the coding manual that MoD should be coded when it is clearly stated in the paper (similarly to BCTTv1). When both coders identified a segment of the text as stating a MoD there was higher agreement about which specific MoD was stated, which demonstrates the utility of the MoD in distinguishing between different MoDs and clearly defining them.

**Step 5: could it be that the lower agreement between raters was not related to the fact that they were less familiar with the ontology, but by the fact that there were was a wider variety in target behaviors in this selection of reports? Taxonomies are also mostly applied to diet, physical activity, addictive behaviours; could it be that the ontology does not fit as well with screening, infectious diseases etc?**

 This is an interesting point. The MoD ontology was designed to be applicable across behaviours, and MoD reporting or lack of it seems to be consistent across behaviours. We hope that future research using this ontology will provides the necessary data to explore this issue further, i.e. if lower agreement are related with familiarity and/or stability across behaviours.

**Table 2 mentions inter-rater reliability twice for step 3: typo?**

 Yes, it was a typo. Thank you for pointing it out.

**Table 3: definition of video game delivery seems to copy-pasted from the level above?**

 Thank you for noticing this. We have now changed the definition to “Electronic mode of delivery that involves the intervention recipient playing a computer game.”

**Table 3: Somatic alteration mode of delivery - also typo (copy-paste above)?**

 Again, thank you for spotting this typo. We have changed to “Mode of delivery that involves modifying the structure of the body of the recipient of the intervention”

# Peer review: Reviewer response for version 1

**DOI:** 10.21956/wellcomeopenres.17447.r39010

**Published:** 2020-07-15

**Authors:** Lucie Byrne-Davis

**Affiliations:** 1 Division of Medical Education, University of Manchester, Manchester, UK

This paper presents the development of an ontology of 'modes of delivery' of behaviour change interventions. It is one of the studies from the Human Behaviour Change Project and is a welcome addition to the literature. Overall, the paper is very well written and the studies sound. My only issue is about the extent to which the introduction includes references to other taxonomies/ontologies beyond the three that it does mention, and therefore how the paper is situated in the literature both  in the introduction and discussion sections.

 Abstract

 I didn't understand the sentence "Relationships between entities consist of *is_a*."

 Should the conclusion in the abstract recommend that people should be familiar with the ontology to ensure that it was used reliably, given that the reliability was only 0.58 when they were unfamiliar?

 Introduction

 You introduce three classification systems but then move straight into the BCTTv1. It is not clear why you focus on that one and so this paragraph seems to come from nowhere. Could you make the reason you are moving from the three systems to the BCTTv1 more obvious? Also, you start a new paragraph after introducing the three systems but that is a very short paragraph, so I would suggest this needs to be one paragraph together. I also expected in the introduction to see more reference to previous taxonomies and problematising these to establish why this ontology was so important. You don't, for example, mention the EPOC taxonomy and I was not sure why.

 Methods and results

 Step 1. This step specifies health behaviours. Previously, you have not specified that this relates to health behaviours specifically, in fact you introduce this as including environmental and social problems, and some of the earlier work is related to health worker behaviours. It would be good to have some clarity about whether this is all human behaviour (which I think it is) and to what extent the methods relied on interventions related to health behaviours and whether this is a limitation of the methods. I know you do state this as a limitation but it would be good to see this up front. in the methods and a rationale for why the study was conducted in this way.

 Step 2. Can you report the response rate (either in methods or results) and where the raters were from. I'm particularly interested in whether all were from a particular part of the world, what institutions were included. Much of the work rests on these individuals being experts so I think it would be appropriate to include some further  information in the text that summarises their credentials and any potential biases they might introduce into the initial ontology.

 Discussion

 As per the introduction, it would be useful to see how this ontology fits with previous attempts at classifying modes of delivery. If there are none (if the EPOC taxonomy is not an example of this) then it would be good to state that as part of the reason for developing this anew.

Is the work clearly and accurately presented and does it cite the current literature?

Partly

If applicable, is the statistical analysis and its interpretation appropriate?

Yes

Are all the source data underlying the results available to ensure full reproducibility?

Yes

Is the study design appropriate and is the work technically sound?

Yes

Are the conclusions drawn adequately supported by the results?

Yes

Are sufficient details of methods and analysis provided to allow replication by others?

Yes

Reviewer Expertise:

Behaviour change in health settings, particularly  health worker practice change; health worker education

I confirm that I have read this submission and believe that I have an appropriate level of expertise to confirm that it is of an acceptable scientific standard, however I have significant reservations, as outlined above.

# Response

**Published:** 2021-02-15

**Authors:** Marta Marques

**Affiliations:** Trinity College Dublin, Ireland

**This paper presents the development of an ontology of 'modes of delivery' of behaviour change interventions. It is one of the studies from the Human Behaviour Change Project and is a welcome addition to the literature. Overall, the paper is very well written and the studies sound. My only issue is about the extent to which the introduction includes references to other taxonomies/ontologies beyond the three that it does mention, and therefore how the paper is situated in the literature both  in the introduction and discussion sections.**

 We appreciate the reviewer’ positive feedback and addressed each suggestion and comment.

**Abstract: I didn't understand the sentence "Relationships between entities consist of *is_a*."**

 We have changed the sentence to “Relationships between entities are hierarchical e.g. “Face-to-face mode of delivery is_a human interactional mode of delivery”

**Abstract: Should the conclusion in the abstract recommend that people should be familiar with the ontology to ensure that it was used reliably, given that the reliability was only 0.58 when they were unfamiliar?**

 We understand the reviewer concern. The recommendation for this and the other HBCP ontologies is that anyone interested in using it, especially for formal coding exercises should first familiarise themselves with it. We have added a sentence to the discussion section of the manuscript as follows:  “It is our recommendation that anyone interested in using the MoD ontology should first familiarise themselves with the MoD entities (labels, definitions and examples) and their relationships, read the coding manual, and conduct some trial annotation and assessment of reliability.”

**Introduction**

**You introduce three classification systems but then move straight into the BCTTv1. It is not clear why you focus on that one and so this paragraph seems to come from nowhere. Could you make the reason you are moving from the three systems to the BCTTv1 more obvious? Also, you start a new paragraph after introducing the three systems but that is a very short paragraph, so I would suggest this needs to be one paragraph together. I also expected in the introduction to see more reference to previous taxonomies and problematising these to establish why this ontology was so important. You don't, for example, mention the EPOC taxonomy and I was not sure why.**

 Thank you for your comment. We have revised this section to reflect the BCTTv1 as an example of a taxonomy focusing on the content of interventions. In addition, we added information about the EPOC taxonomy in the “Delivery of Behaviour Change Interventions” section.

**Methods and results**

**Step 1. This step specifies health behaviours. Previously, you have not specified that this relates to health behaviours specifically, in fact you introduce this as including environmental and social problems, and some of the earlier work is related to health worker behaviours. It would be good to have some clarity about whether this is all human behaviour (which I think it is) and to what extent the methods relied on interventions related to health behaviours and whether this is a limitation of the methods. I know you do state this as a limitation but it would be good to see this up front. in the methods and a rationale for why the study was conducted in this way.**

 Thank you for pointing this out. This is indeed intended as an ontology of modes of delivery for all domains of behaviour change interventions. The limitations section of the discussion addresses the limitations of having annotated mainly health-related behaviour papers within the ontology development stages, and we have now made this point clearer, as follows: “Secondly, the intervention reports included in the annotation process were from two larger projects, the Theory and Techniques Project ( Michie *et al.*, 2018) and the Human Behaviour-Change Project ( Michie *et al.*, 2017). The intervention reports annotated within the ontology development mainly addressed two health-related behaviours, smoking cessation and physical activity; there is always the possibility that other literature within and outside the health domain may indicate modes of delivery not captured in our set of papers or by our group of experts. However, external inter-rater reliability was tested across diverse behaviours and found to be acceptable. Future applications of the ontologies to a wider collection of non-health related behaviours and contexts is likely to extend and improve the ontology.”

**Step 2. Can you report the response rate (either in methods or results) and where the raters were from. I'm particularly interested in whether all were from a particular part of the world, what institutions were included. Much of the work rests on these individuals being experts so I think it would be appropriate to include some further  information in the text that summarises their credentials and any potential biases they might introduce into the initial ontology.**

 We thank the reviewer for this important point. We have data on the type of organisations reviewers were from: 18 were from universities, 5 from commercial sector organisations, 1 from public sector organisations and 1 third sector; and the countries: 12 experts were from the United Kingdom, 2 from the United States of America, 3 from Ireland, 1 from Canada, 1 from the Netherlands, 1 from New Zealand, and for 5 of them we have no information about the country.  We also have a list of the specific institutions they were from. We don’t have response rate data as the invitations to participate were sent out via third-party mailing lists (e.g. conference) and so we do not know how many people were subscribed to each list. We have added the following information to the results, step 2: “Feedback on the MoD ontology through the open review feedback form was received by 25 experts, of which 18 were from universities, 5 were from commercial sector organisations, 1 from public sector organisations and 1 from third sector. Twelve experts were from the United Kingdom, 2 from the United States of America, 3 from Ireland, 1 from Canada, 1 from the Netherlands, 1 from New Zealand, and we have no information about the country for the remaining 5 experts.”

**Discussion**

**As per the introduction, it would be useful to see how this ontology fits with previous attempts at classifying modes of delivery. If there are none (if the EPOC taxonomy is not an example of this) then it would be good to state that as part of the reason for developing this.**

 Thank you for this comment. We have addressed this comment in the introduction in “Delivery of Behaviour change interventions”. Further we added a sentence in the discussion to reflect this as follows “Given the lack of classification systems providing comprehensive coverage of how behaviour change interventions and techniques are delivered, we developed the first ontology of modes of delivery (MoD).”
